# Construction of Monitoring Model and Algorithm Design on Passenger Security during Shipping Based on Improved Bayesian Network

**DOI:** 10.1155/2014/158652

**Published:** 2014-08-28

**Authors:** Jiali Wang, Qingnian Zhang, Wenfeng Ji

**Affiliations:** ^1^School of Transportation, Wuhan University of Technology, Wuhan 430063, China; ^2^School of Business, Jianghan University, Wuhan 430058, China; ^3^College of Engineering, Huazhong Agricultural University, Wuhan 430070, China

## Abstract

A large number of data is needed by the computation of the objective Bayesian network, but the data is hard to get in actual computation. The calculation method of Bayesian network was improved in this paper, and the fuzzy-precise Bayesian network was obtained. Then, the fuzzy-precise Bayesian network was used to reason Bayesian network model when the data is limited. The security of passengers during shipping is affected by various factors, and it is hard to predict and control. The index system that has the impact on the passenger safety during shipping was established on basis of the multifield coupling theory in this paper. Meanwhile, the fuzzy-precise Bayesian network was applied to monitor the security of passengers in the shipping process. The model was applied to monitor the passenger safety during shipping of a shipping company in Hainan, and the effectiveness of this model was examined. This research work provides guidance for guaranteeing security of passengers during shipping.

## 1. Introduction

The South Korean ferry “Sewol” carrying 476 passengers sank off South Korea's southwest coast on April, 16, 2014, causing hundreds of deaths and miss. This accident sounded the alarm of passenger security during shipping all over the world. Nowadays, the time-consuming shipping transportation is mainly used for tourism purpose instead of travel purpose. A safe and comfortable journey is the common expectation of tourists. Shipping management department and shipping enterprises always pay high attention to passenger security during shipping process. However, many factors have influence on shipping safety, thus making the passenger security during shipping difficult to be predicted and controlled [1]. Only few researches on passenger security have been reported, most of which only focus on the safety of carriers but neglect the characteristics of passengers [2]. The passenger security based on characteristics of passengers and transportation carriers was studied by improving the computing method of Bayesian network in this paper, and the “fuzzy-accurate Bayesian network” was established to evaluate passenger security during shipping [3, 4].

Bayesian network is an inference network model based on the uncertainty and variability of probability and is applicable to explore various uncertainty and probability problems. When Bayesian network was used in decision-making events involving various control factors, it can make correct inferences from incomplete, ambiguous, or uncertain knowledge or information. Therefore, it is widely used in security evaluation. Both subjective Bayesian network and objective Bayesian network are applicable to security evaluation. Subjective Bayesian network uses the Bayesian network model to predict the probability of accident occurrence based on expert's subjective estimation result when appropriate objective data are unavailable. The subjective Bayesian network also can be called fuzzy Bayesian network because it predicts the probability of even occurrence through fuzzy set theory. Objective Bayesian network makes network inferences on the probability of the accident occurrence which happened based on collected abundant relevant node data. When using Bayesian network to evaluate passenger security during shipping, objective data are difficult to be collected because of various influencing factors and incomplete records of shipping enterprises of passenger transport. Although the author has collected some objective data about the passenger security during shipping, these data cannot cover all influencing factors [5]. Therefore, the established Bayesian network model failed to predict the probability of accident occurrence under all conditions and presented many unreasonable predictions. The modeling process of both subjective and objective Bayesian network was improved in this paper, and the “fuzzy-accurate Bayesian network” was established. This “fuzzy-accurate Bayesian network” not only can predict probability of accident occurrence under all conditions, but also takes the maximum use of collected objective data; thus it is more applicable to be studied on prediction of passenger security during shipping [6, 7].

## 2. Fuzzy-Accurate Bayesian Network

Bayesian network, also known as Belief Network, was developed from the Bayes method proposed by Judea Peral in 1988. It has been widely used in fault diagnosis, data mining, medical diagnosis, and traffic safety in considering its unique uncertainty, knowledge representation form, strong probability, expressive ability, and the incremental learning of comprehensive priori knowledge. In particular, Bayesian network has achieved outstanding success in traffic safety field, such as causing analysis of traffic disasters, early-warning of traffic safety, and traffic safety evaluation [8].

The main theoretical basis of Bayesian network is the Bayes formula, known as the posterior probability formula. Suppose the prior probability is *p*(*B*_*i*_) and *p*(*A*_*j*_∣*B*_*i*_)  (*i* = 1,2,…, *n*, *j* = 1,2,…, *m*) is known; then the posterior probability calculated from the Bayes formula is [9, 10]

(1) $$\begin{array}{c} P\left(B_{i}\;|\;A_{j}\right)=\frac{P\left(B_{i}\right)P\left(A_{j}\;|\;B_{i}\right)}{\sum_{k=1}^{m}P\left(B_{i}\right)P\left(A_{k}\;|\;B_{i}\right)}. \end{array}$$

**Figure 1:**
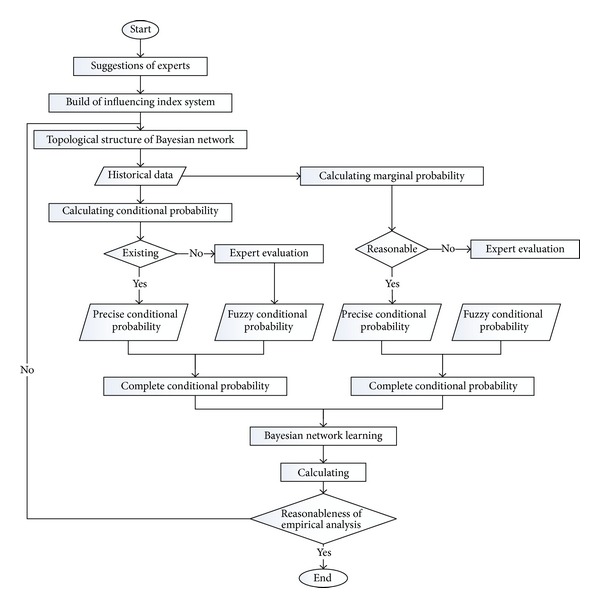
Topological graph of calculation process of the fuzzy-accurate Bayesian network.

In this paper, a fuzzy-accurate Bayesian network was established by combing the objective Bayesian network and fuzzy Bayesian network, which was used to monitor passenger security during shipping.  Figure 1 shows its calculation flow chart. The established fuzzy-accurate Bayesian network mainly accomplishes Bayesian network computation tasks when no adequate historical data are available [11, 12].

Fuzzy set theory is essential to perfect the data structure of the fuzzy-accurate Bayesian network. Fuzzy set, the set of specific-property objects with ambiguous limits or boundaries, can represent expert's evaluation results more intuitively when there is no specific data available. The fuzzy language description, corresponding fuzzy number, and *λ*-cut set are listed in Table 1.

Generally speaking, it has to take the evaluation results of several experts into account when quantifying the probability of occurrence of a certain accident. Therefore, the evaluation results of several experts were integrated by using the arithmetic method in this paper. The comprehensive evaluation of *n* experts can be expressed as

(2) $$\begin{array}{c} P\left(i\right)=\frac{f_{i1}⊕f_{i2}⊕\cdots ⊕f_{im}}{n},\;i=1,2,\ldots ,m, \end{array}$$

where *P*_*i*_ is the fuzzy probability of occurrence of *i*th accident, *f*_*ij*_ is the fuzzy number of *j*th expert to the *i*th accident, and *m* is the amount of accidents [13].

The fuzzy evaluation results of several experts were processed by integral method in this paper. Suppose *P* is the fuzzy number of L-R type; the ambiguity resolution of *P* is

(3) $$\begin{array}{c} I\left(P\right)=\left(1-\varepsilon \right)I_{\text{R}}\left(P\right)+\varepsilon I_{\text{L}}\left(P\right), \end{array}$$

where *ε* ∈ [0,1] is the optimistic coefficient. When *ε* = 0 and *ε* = 1, *I*(*P*) are the upper and lower limits of the ambiguity resolution of *P*. When *ε* = 0.5, *I*(*P*) is the representative value of ambiguity resolution of *P*. *I*_R_(*P*) and *I*_L_(*P*) are the integral values of the right and left inverse membership functions of the fuzzy number. For the triangle fuzzy number, *I*_R_(*P*) and *I*_L_(*P*) can be expressed by *λ*-cut set:

(4) $$\begin{array}{c} I_{\text{R}}\left(p\right)\frac{1}{2}\left(\overset{1}{\underset{i=0.1}{\sum }}\lambda_{\text{R}}\left(p\right)\Delta \lambda +\overset{0.9}{\underset{i=0}{\sum }}\lambda_{\text{R}}\left(p\right)\Delta \lambda \right),\\ I_{\text{L}}\left(p\right)\frac{1}{2}\left(\overset{1}{\underset{i=0.1}{\sum }}\lambda_{\text{L}}\left(p\right)\Delta \lambda +\overset{0.9}{\underset{i=0}{\sum }}\lambda_{\text{L}}\left(p\right)\Delta \lambda \right), \end{array}$$

where *λ*_R_(*P*) and *λ*_L_(*P*) are the upper and lower limits of the *λ*-cut set of *P*. Consider *λ* = 0, 0.1, 0.2,…, 1;  Δ*λ* = 0.1 [14, 15].

## 3. Determination of Evaluation Index System and Its Topological Structure for Passenger Security during Shipping

Influencing factors of passenger security during shipping were analyzed from five aspects (human, machine, environment, management system, and characteristics of passengers) by using the theory of “multifield coupling” in physics. An influencing index system of passenger security during shipping was established, and a total 50 influencing factors were got [16, 17]. However, so many influencing factors, the complicated network model and difficult data acquisition, make the probability of accident occurrence of a certain node difficult to be estimated by using the fuzzy language. Hence, the weight of influencing indexes on passenger security during shipping was calculated and ranked through network analysis method in this paper. A total of 26 important indexes were selected (Table 2).

**Figure 2:**
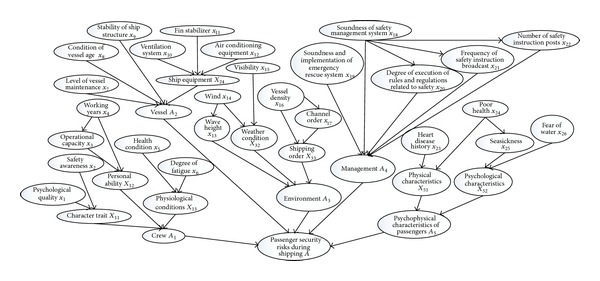
Bayesian network topology of passenger security monitoring during shipping.

The effect of evaluation indexes in Table 2 on the passenger security during shipping was analyzed. Suggested by experts, the Bayesian network topology of passenger security monitoring during shipping was established (Figure 2).

These influencing factors can only be used after being transferred into level-based values, that is, determining their node domain. This was not introduced in this paper due to the paper length limitation.

## 4. Construction of Monitoring Model of Passenger Security during Shipping by Fuzzy-Precise Bayesian Network

### 4.1. Bayesian Network of Passenger Security during Shipping Based on Historical Data

The historical sample data are mainly related to passenger accident risks on 16 passenger ships of Hainan HH Shipping Company in 2012 and 2013. The relevant data of 1,500 passengers was collected, and 1286 data was effective. Since these data could not cover all nodes' conditions, they shall be reasoned by Bayesian network firstly to identify influencing factors of passenger security of evidence nodes. Then, the marginal probability of the evidence nodes as well as the conditional probabilities of intermediate nodes and target nodes that influence the passenger security during shipping can be acquired.

**Figure 3:**
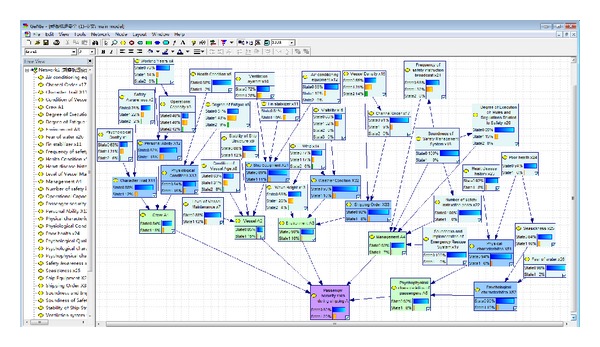
GeNIe simulation of Bayesian network based on historical data.

During the Bayesian network inference process, the software of GeNIe for Bayesian network modeling was used in this paper. The GeNIe was developed by the decision-making system laboratory of University of Pittsburgh providing development environment for imaging decision-making theoretical model and can be used for project study or even business field. It not only has visual windows, but also can make accurate and approximate inferences as well as parameter and structural learning, thus establishing static and dynamic Bayesian network models. The cleared historical data was input into the established GeNIe Bayesian network model to calculate the marginal probability and conditional probability of the Bayesian network, and the passenger security during shipping was evaluated (Figure 3).

Figure 3 presents a generally high passenger security during shipping. The probability of passenger security during shipping is 0.80, while the probability of accident occurrence is 0.20.

The marginal probability of evidence nodes of influencing factors on passenger security was calculated (Table 3). The conditional probabilities of evidence nodes were calculated based on maximum posterior estimation (Table 4). It is concluded that there are five influencing factors involved in the marginal probability and 20 influencing factors involved in the conditional probability. Not all conditional probabilities were introduced in this paper due to the limited paper length.

### 4.2. Determination of Marginal Probability and Conditional Probability of Passenger Security Nodes Based on Fuzzy Set Theory

The collected historical data only involves limited passenger ships, a small navigation geographic reach, and only one company's management system, thus resulting in the poor accuracy of marginal probability concerning ship, environment, and management. Therefore, these absent and unreal data have to be revised by using Delphi method and fuzzy set theory. On the contrary, the marginal probability concerning characteristics of seamen and travelers is believed reliable since the historical data involves adequate samples with certain representativeness. But the historical data size is too small to cover all conditions of evidence nodes, indicating the incompleteness of conditional probability and poor accuracy of passenger security during shipping calculated from the established Bayesian network. Therefore, the conditional probability of nodes beyond the historical data shall be estimated.

10 experts (3 researchers of passenger ships, 3 managers of passenger ship, and 4 senior captains) were invited to correct the unreal marginal probability (Table 5) by using fuzzy language and predict conditional probability (Table 6) of nodes beyond the collected historical data.

### 4.3. Bayesian Network Inference of Passenger Security during Shipping under Complete Data

Based on the established hierarchical structure of the Bayesian network, and calculated marginal probability and conditional probability of evidence nodes, the passenger security during shipping can be reasoned by using the joint probability distribution. The probability of “passenger security during shipping” of target nodes was calculated directly by GeNIE (Table 7).

## 5. Empirical Study

To test the feasibility and validity of the research result, the established fuzzy-accurate Bayesian network was used on a passenger ship to monitor the passenger security of the lane from the Haikou to the Hai'an in Qiongzhou Strait, China. Parameters of the testing vessel: length was 88.4 m, weight was 3,840 tons, motor of main engine was 1103 kw × 2, width was 16 m, and draft was 3.4 m. The manufacturing time was January, 2002, its rated passenger capacity was 648 people, rated vehicle loads was 40 vehicles, and seamen in the ship were 21 (including 8 senior seamen). It carried 522 passengers from the Hai'an Port of Zhanjiang to Xiuying Port of Haikou on 10:00 on August, 4, 2013. The weather condition was as follows: a cloudy day with showers, southeast wind 3-4, 1 m high waves, and visibility within 1500 km. Three passengers on the ship were selected randomly for questionnaire survey of psychophysical characteristics, and their health conditions at the destination were observed.

The monitoring data were converted into status value of evidence nodes of the Bayesian network according to the evaluation standard. Unmonitored node states were defaulted to normal. The status values of different evidence nodes are listed in Tables 8 and 9.

The evidence states in Table 9 were inputted into the fuzzy-accurate Bayesian network model established by GeNIe to calculate the joint probability of root nodes (Tables 10, 11, and 12).

Tables 10, 11, and 12 list the overall safety probability and safety probability of five subindexes (seaman, ship, environment, management, and psychophysical characteristics) of three sample passengers. It can be concluded that Passenger 3 has the highest security risk during the trip, followed by Passenger 2 and Passenger 1 successively. Compared to the probability of accident occurrence under general conditions (0.1027, Table 7), Passenger 2 and Passenger 3 had higher probability of accident risk and need to be pay more attention. In this experiment, a follow-up survey was conducted to these three passengers and found out that all of them have seasickness, which influenced the passenger security during shipping.

## 6. Conclusions

Shipping accidents frequently occurred, and these accidents are caused by various factors, thus making it difficult to predict passenger security during shipping. The “fuzzy-accurate Bayesian network” theory was used to establish a model for monitoring passenger security during shipping. The fuzzy-accurate Bayesian network was obtained by improving the computation process of Bayesian network. It can offset the poor performance of Bayesian network caused by the difficult data acquisition and failure to cover all influencing factors of passenger security during shipping. A model used to monitor passenger security during shipping was established based on the Bayesian network inference from the established influencing index system and its topological structure, historical data, and prior probability on basis of fuzzy set theory. This monitoring model verified the validity through an empirical study and can be used to predict passenger security in a certain period of coming ship trip.

## Figures and Tables

**Table 1:** Fuzzy number and *λ*-cut set.

Language description	Fuzzy number	*λ*-Cut set
Very high (VH)	*f*_VH_ = (0.8, 0.9, 1.0)	*f*_VH_^*λ*^ = [0.1*λ* + 0.8, −0.1*λ* + 1]
High (H)	*f*_H_ = (0.7, 0.8, 0.9)	*f*_H_^*λ*^ = [0.1*λ* + 0.7, −0.1*λ* + 0.9]
Fairly high (FH)	*f*_FH_ = (0.5, 0.6, 0.7, 0.8)	*f*_FH_^*λ*^ = [0.1*λ* + 0.5, −0.1*λ* + 0.8]
Moderate (M)	*f*_M_ = (0.4, 0.5, 0.6)	*f*_M_^*λ*^ = [0.1*λ* + 0.4, −0.1*λ* + 0.6]
Fairly low (FL)	*f*_FL_ = (0.2, 0.3, 0.4, 0.5)	*f*_FL_^*λ*^ = [0.1*λ* + 0.2, −0.1*λ* + 0.5]
Low (L)	*f*_L_ = (0.1, 0.2, 0.3)	*f*_L_^*λ*^ = [0.1*λ* + 0.1, −0.1*λ* + 0.3]
Very low (VL)	*f*_VL_ = (0.0, 0.1, 0.2)	*f*_VL_^*λ*^ = [0.1*λ* + 0, −0.1*λ* + 0.2]

**Table 2:** Influencing index system of passenger security during shipping.

Target layer	System layer	Criterion layer	Factor layer
Passenger security risks during shipping *A*	Crew *A*_1_	Character trait *X*_11_	Psychological quality *x*_1_
			Safety awareness *x*_2_
		Personal ability *X*_12_	Operational capacity *x*_3_
			Working years *x*_4_
		Physiological conditions *X*_13_	Health condition *x*_5_
			Degree of fatigue *x*_6_
	Vessel *A*_2_	Vessel maintenance *X*_21_	Level of vessel maintenance *x*_7_
		Vessel age *X*_22_	Condition of vessel age *x*_8_
		Hull structure *X*_23_	Stability of ship structure *x*_9_
		Ship equipment *X*_24_	Ventilation system *x*_10_
			Fin stabilizer *x*_11_
			Air conditioning equipment *x*_12_
	Environment *A*_3_	Hydrologic condition *X*_31_	Wave height *x*_13_
		Weather condition *X*_32_	Wind *x*_14_
			Visibility *x*_15_
		Shipping order *X*_33_	Vessel density *x*_16_
			Channel order *x*_17_
	Management *A*_4_	Safety management system *X*_41_	Soundness of safety management system *x*_18_
		Emergency rescue system *X*_42_	Soundness and implementation of emergency rescue System *x*_19_
		Execution of rules and regulations related to safety *X*_43_	Degree of execution of rules and regulations related to safety *x*_20_
		Safety instruction broadcast *X*_44_	Frequency of safety instruction broadcast *x*_21_
		Safety instruction post *X*_45_	Number of safety instruction posts *x*_22_
	Psychophysical characteristics of passengers *A*_5_	Physical characteristics *X*_51_	Heart disease history *x*_23_
			Poor health *x*_24_
		Psychological characteristics *X*_52_	Seasickness *x*_25_
			Fear of water *x*_26_

**Table 3:** Marginal probability of evidence nodes.

Psychological quality *x*_1_		Safety awareness *x*_2_		Length of service on ships *x*_4_		Health condition *x*_5_		Degree of fatigue *x*_6_	
Level	Marginal probability	Level	Marginal probability	Level	Marginal probability	Level	Marginal probability	Level	Marginal probability
1	0.63	1	0.76	1	0.78	1	0.98	1	0.51
2	0.31	2	0.22	2	0.14	2	0.02	2	0.40
3	0.06	3	0.02	3	0.08			3	0.09

**Table 4:** Conditional probability of intermediate node (personality characteristics).

Indexes related to “personality characteristics”		Evaluation level of “personality characteristics”	
Psychological quality *x*_1_	Safety awareness *x*_2_	1	2
1	1	1	0
1	2	0.82	0.18
2	1	0.86	0.14
2	2	0.67	0.33
1	3	0.36	0.64
3	1	0.50	0.50

**Table 5:** Marginal probability of environment's evidence nodes calculated from fuzzy set.

Wave height		Wind		Visibility		Ship density		Navigation order	
Level	Evaluation standard	Level	Evaluation standard	Level	Evaluation standard	Level	Evaluation standard	Level	Evaluation standard
1	0.76	1	0.83	1	0.75	1	0.66	1	0.82
2	0.19	2	0.13	2	0.19	2	0.25	2	0.11
3	0.05	3	0.04	3	0.04	3	0.09	3	0.07
				4	0.75				

**Table 6:** Conditional probability of the intermediate node (personality of characteristics) beyond the historical data.

Indexes related to “personality characteristics”		Evaluation level of “personality characteristics”	
Psychological quality *x*_1_	Safety awareness *x*_2_	1	2
2	3	0.23	0.77
3	2	0.22	0.78
3	3	0.11	0.89

**Table 7:** Probability of passenger security during shipping and subindexes.

Node	Probability of safety	Probability of accident occurrence
Passenger security risks during shipping *A*	0.8973	0.1027
Crew *A*_1_	0.8341	0.1659
Vessel *A*_2_	0.8197	0.1813
Environment *A*_3_	0.8590	0.1410
Management *A*_4_	0.8953	0.1047
Psychophysical characteristics of passengers *A*_5_	0.9322	0.0678

**Table 8:** Node states of the testing ship at the Qiongzhou Strait.

Crew		Vessel		Environment		Management	
Evidence node	State	Evidence node	State	Evidence node	State	Evidence node	State
*x*_1_	1	*x*_7_	1	*x*_14_	1	*x*_18_	1
*x*_2_	1	*x*_8_	1	*x*_15_	1	*x*_19_	1
*x*_4_	1	*x*_9_	1	*x*_16_	2		
*x*_5_	1	*x*_10_	1				
*x*_6_	1	*x*_11_	1				
		*x*_12_	1				

**Table 9:** Node states of psychophysical characteristics of selected respondents.

Evidence node of passengers' psychophysical characteristics	*x*_23_	*x*_24_	*x*_26_
Passenger 1	1	1	1
Passenger 2	1	2	1
Passenger 3	2	2	1

**Table 10:** Safety probability of Passenger 1.

Node	Probability of safety	Probability of accident occurrence
Passenger security risks during shipping *A*	0.9682	0.0318
Crew *A*_1_	0.9677	0.0323
Vessel *A*_2_	0.9748	0.0252
Environment *A*_3_	0.9419	0.0581
Management *A*_4_	0.9502	0.0498
Psychophysical characteristics of passengers *A*_5_	0.9696	0.0304

**Table 11:** Safety probability of Passenger 2.

Node	Probability of safety	Probability of accident occurrence
Passenger security risks during shipping *A*	0.8796	0.1204
Crew *A*_1_	0.9677	0.0323
Vessel *A*_2_	0.9748	0.0252
Environment *A*_3_	0.9419	0.0581
Management *A*_4_	0.9502	0.0498
Psychophysical characteristics of passengers *A*_5_	0.7608	0.2392

**Table 12:** Safety probability of Passenger 3.

Node	Probability of safety	Probability of accident occurrence
Passenger security risks during shipping *A*	0.7430	0.2570
Crew *A*_1_	0.9677	0.0323
Vessel *A*_2_	0.9748	0.0252
Environment *A*_3_	0.9419	0.0581
Management *A*_4_	0.9502	0.0498
Psychophysical characteristics of passengers *A*_5_	0.4387	0.5613
